# Improved estimates of foetal growth are associated with perinatal outcomes: A latent modelling approach in a population-based birth cohort

**DOI:** 10.7189/jogh.13.04070

**Published:** 2023-09-11

**Authors:** Bárbara H Lourenço, Paulo AR Neves, Marly A Cardoso, Marcia C Castro, MINA-Brazil Study Group, Alicia Matijasevich, Bárbara Hatzlhoffer Lourenço, Maíra Barreto Malta, Marcelo Urbano Ferreira, Marly Augusto Cardoso, Paulo Augusto Ribeiro Neves, Ana Alice Damasceno, Bruno Pereira da Silva, Rodrigo Medeiros de Souza, Simone Ladeia-Andrade, Marcia C Castro

**Affiliations:** 1 Department of Nutrition, School of Public Health, University of São Paulo, São Paulo, Brazil; 2 Department of Global Health and Population, Harvard T.H. Chan School of Public Health, Boston, Massachusetts, USA; 3 Centre for Global Child Health, The Hospital for Sick Children, University of Toronto, Toronto, Canada

## Abstract

**Background:**

We aimed to estimate latent foetal growth conditions and explore their determinants among maternal characteristics and ultrasound biometric parameters. We additionally investigated the influence of foetal growth conditions on perinatal variables.

**Methods:**

We used data from live-born singletons in the Maternal and Child Health and Nutrition in Acre, Brazil (MINA-Brazil Study) population-based birth cohort. Maternal and perinatal characteristics were assessed in medical records from the maternity hospital and interviews with participants from July 2015 to June 2016. A sub-sample went through ultrasound examinations during the antenatal period, with assessment of foetal head and abdominal circumferences, and femur length. We estimated latent foetal growth conditions with a structural equation modelling framework, informed by the child’s birth weight z-scores (BWZ) and birth length z-scores (BLZ) according to gestational age. Odds ratios and 95% confidence intervals (CIs) for the occurrence of perinatal events were estimated according to linear predictions of the latent variable.

**Results:**

We included 1253 participants. Latent foetal growth conditions explained 88.3% of BWZ and 53.7% of BLZ variation. Maternal elevated blood pressure, primiparity, smoking, malaria, and insufficient gestational weight gain negatively impacted foetal growth conditions. In the subsample (n = 499), ultrasound biometric parameters assessed at 28 weeks were positively associated with the latent variable, with the largest contribution from foetal abdominal circumference. Each standardised unit of predicted foetal growth conditions halved the chance for preterm birth (95% CI = 0.26, 0.74) and longer hospital stay (>3 days) (95% CI = 0.28, 0.88). Conversely, BWZ and BLZ were not independently associated with these perinatal variables in separate logistic regression models.

**Conclusions:**

Latent foetal growth conditions jointly encompassing weight gain and linear growth during gestation were negatively influenced by a scenario of dual burden of maternal morbidities, with perinatal implications.

Foetal growth conditions impact a valuable window of opportunity for the promotion of a child’s healthy development in the first years of life [1]. Birth weight and length according to gestational age (GA) [2] result from sequential hyperplastic and hypertrophic processes in an underlying intrauterine environment and are main indicators of these conditions. In pooled analyses of longitudinal studies, the interval between birth and three months of life accounted for the highest incidence of stunting and wasting among children from South Asia, sub-Saharan Africa, and Latin America [3,4]. Such a critical period must be addressed with interventions devised since the antenatal period [5].

Modifiable risk factors for foetal growth constraint are particularly important in low-resource settings worldwide, which face concurrent burdens of non-communicable and infectious diseases [6]. For instance, in a birth cohort in the Brazilian Amazon [7], we have observed frequent patterns of inadequate gestational weight gain (GWG) [8] in association with blood pressure levels in pregnant women [9]. Living in a malaria endemic area, participants affected mostly by *Plasmodium vivax* infections during pregnancy delivered babies with significantly lower birth weight and length [10].

Nevertheless, there is a lack of real-time accurate methods to assess the intrauterine environment. Describing foetal growth conditions as a latent variable that mediates the influence of maternal traits on newborn size may advance the management of factors that coalesce distinct components of offspring development during gestation. Previous studies in the Philippines and in rural regions of the USA have supported this approach [11,12], but considered a limited set of predictors of foetal growth conditions, explaining only 8-11% of the variation of the latent variable. The contribution of ultrasound biometric parameters [13] could pinpoint relevant components of intrauterine physical growth, but remains understudied. Similarly, evidence on the association of latent foetal growth conditions with perinatal variables is currently not available.

To address these gaps, we hypothesised that the prediction of latent foetal growth conditions could be greatly improved with additional data on maternal health status and ultrasound biometry. We also assumed that the predicted conditions will be positively associated with perinatal characteristics, and at a larger magnitude if compared to isolated components of newborn size.

Thus, we aimed to expand existing studies on determinants of latent foetal growth conditions in a scenario of dual burden of maternal chronic and infectious morbidities, and analyse the influence of these conditions on critical perinatal variables. In regions comprising the largest share of children affected by various forms of malnutrition [14], improving the comprehension and measurement of the underlying conditions that shape newborn size could inform strategies focused on the integration of pregnancy and paediatric care [15], paving the way for better short and long-term health outcomes.

## METHODS

### Study design and population

We based this exploratory study of latent foetal growth conditions on baseline assessment data from the Maternal and Child Health and Nutrition in Acre, Brazil (MINA-BRAZIL) population-based birth cohort, established in Cruzeiro do Sul, Brazilian Amazon [7]. With an estimated 81 519 inhabitants in 2015 [16], the municipality has poor antenatal care indicators amid socioeconomic and health disparities, and is located in a malaria hotspot in Brazil [10,17].

The research team screened medical records of birth-related admissions from July 2015 to June 2016 on a daily basis, at the only maternity hospital in the municipality, where 96% of all deliveries take place. They subsequently interviewed participants during their hospital stay on sociodemographic, health, and obstetric characteristics [7]. The MINA-Brazil Study also held antenatal assessments in a subsample of pregnant women who were referred with up to 20 gestational weeks for antenatal care in primary health care units of the urban area. Enrolled from February 2015 to January 2016, these subjects reported living in the city and having the intention to give birth in the local maternity hospital. They participated in prospective clinical evaluations during pregnancy, including ultrasound examinations [7,18].

From 1881 births, there were 112 abortions and 16 stillbirths; out of the 1753 live births, 184 mothers refused participation, and 18 were not assessed before hospital discharge. Of these, we recovered the data from 12 participants from the evaluations conducted by the study team during pregnancy. We further excluded 26 twins from this analysis. Therefore, 1537 live-born singletons were eligible, with information on newborn size available for 99% of them (n = 1516). Complete data on maternal characteristics were available for 82% of eligible participants (n = 1253). A subsample of 499 participants (40% of the total) had additional ultrasound data from the antenatal period (Figure S1 in the **Online Supplementary Document**).

The Institutional Review Board of the School of Public Health, University of São Paulo (protocol 872.613/2014) approved the study procedures. We obtained written informed consent from all participants; for mothers aged <18 years, their legal guardian provided consent.

### Measures

Trained research assistants conducted interviews with participants within 12 hours after delivery, collecting information on age, skin colour (categorised as white or non-white (mulatto/black/yellow/indigenous)), ownership of household assets (generating a wealth index [19], in terciles), schooling (≤9 or >9 years), assistance from the *Bolsa Família* conditional cash transfer program (no or yes), and living with a partner (no or yes). Assessment of the occurrence of pre-pregnancy morbidities included elevated blood pressure, diabetes, and malaria; participants also reported on previous pregnancies and planning of the current pregnancy (no or yes).

We retrieved information from each participant’s antenatal card on the number of antenatal care visits (<6 or ≥6), nutritional supplement use (no or yes), smoking during pregnancy (no or yes), maternal height (metres), and pre-pregnancy weight (kilograms). Pre-pregnancy weight was ascertained before 14 weeks or self-reported if the first antenatal visit occurred afterward. Information in these cards had a satisfactory agreement with standardised anthropometry procedures performed by the research team in the subsample followed-up during pregnancy [20]. We classified pre-pregnancy nutritional status according to body mass index (BMI) [21]. Malaria episodes during pregnancy were retrospectively obtained from the Malaria Epidemiological Surveillance and Information System of the Brazilian Ministry of Health [22].

We collected pre-delivery maternal weight (in kilograms) and GA (in weeks) from medical records at the maternity hospital. We calculated total GWG as the difference between pre-delivery and pre-pregnancy weight, categorising them as insufficient (no or yes) according to the Institute of Medicine criteria based on pre-pregnancy BMI [23]. Information on GA from medical records had an acceptable agreement with ultrasound-confirmed estimates in the subsample [18].

We also retrieved data on child’s sex, birth weight (in kilograms), and length (in centimetres) from medical records. Research assistants trained hospital staff involved in newborn care on standardised anthropometric procedures; the accuracy of digital scales was checked routinely [7]. We calculated z-scores for birth weight and length (BWZ and BLZ, respectively) for GA and sex, according to the INTERGROWTH-21^st^ Project international standards [2].

Lastly, we gathered information on perinatal variables, including the occurrence of preterm birth (<37 gestational weeks) [24,25], reanimation procedures after birth, infant formula prescription, and duration of hospital stay >3 days [26] (no or yes).

### Subsample ultrasound examinations

Trained field physicians used a portable SonoSite TITAN machine (SonoSite Inc., Bothell, WA) to perform ultrasound examinations in the subsample followed-up during pregnancy [7]. All images were captured following self-scoring quality criteria [27] for the placement of calipers and ellipses with proper visualisation of landmarks in all foetal planes [28]. Blinded re-evaluation of images by an independent expert obstetrician for external quality control indicated >94% of satisfactorily acquired images in all planes [18]. Ultrasound biometric parameters included head circumference (HC), abdominal circumference (AC), and femur length (FL), represented in z-scores for GA considering the INTERGROWTH-21^st^ Project standards [13].

### Statistical analysis

We described the distribution of general characteristics and ultrasound biometric parameters in proportions or medians and interquartile ranges (IQRs). We compared categorical and continuous variable’s using χ^2^ and Mann-Whitney tests, respectively, according to participation in assessments during pregnancy.

**Figure 1:**
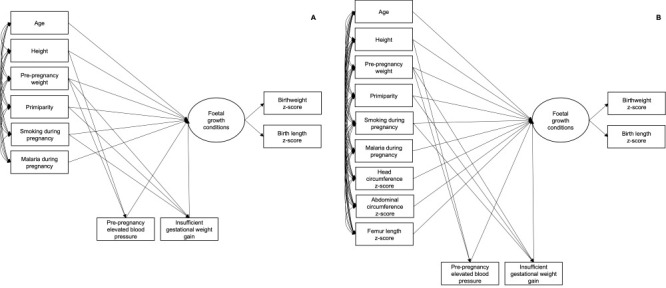
Model representation for latent foetal growth conditions in the MINA-Brazil Study. **Panel A.** Model representation for the total study population. **Panel B.** Model representation for the subsample with ultrasound data.

Using a structural equation modelling (SEM) framework [11], we investigated determinants of latent foetal growth conditions by fitting a multiple indicators multiple causes (MIMIC) model [29] (**Figure 1**). A latent variable is not objectively observed, but rather deduced from covariances between its indicator variables; as per SEM notation, it is represented by an oval in the diagram, while the observed variables are contained in rectangles. The MIMIC model is simultaneously composed of measurement and structural portions [29].

In the measurement model, BWZ and BLZ were depicted as indicators of a continuous latent variable. BWZ was constrained to 1, scaling the latent variable in z-scores. The structural model specified determinants of foetal growth conditions, and variable selection considered their conceptual relevance. We could not confirm the normality assumption for all observed data, as assessed using the Shapiro-Wilk test. Thus, we estimated models using maximum likelihood with Satorra-Bentler scaled χ^2^ statistic, which encompasses a function of fourth-order moments to adjust the standard goodness-of-fit statistics, with corresponding standard errors that are robust to non-normality [29]. We allowed determinants to correlate and additional paths were indicated to pre-pregnancy elevated blood pressure and insufficient GWG. We additionally investigated the effects of ultrasound biometric parameters on the latent variable in the subsample, while disentangling the contributions of HC, AC, and FL.

We assumed the conceptual suitability for a latent variable, since foetal growth conditions may not be directly ascertained. Also, intrauterine weight and length accrual could be addressed as an integrated process under the latent variable. We also assured the statistical suitability of models with latent foetal growth conditions (**Figure 1**) in comparison to alternative models specifying direct effects of antenatal characteristics on BWZ and BLZ, separately (Figure S2 in the **Online Supplementary Document**). We checked for model fit by observing a non-significant χ^2^ statistic and considering the following measures: root mean squared error of approximation (RMSEA) (with a lower bound ideally <0.05), Bayesian information criterion (BIC) (smaller values indicating better fit), and the comparative fit index (CFI) and the Tucker-Lewis index (TLI) (best fit if closer to 1), with Satorra-Bentler adjustments [11,29] (Table S1 in the **Online Supplementary Document**). These statistics consistently favoured the adoption of the approach with a latent variable for foetal growth conditions, which was a more parsimonious model. There was excellent fit to the data, with lower RMSEA and BIC values, and CFI and TLI closer to their ideal fit of 1, corroborating that the indicators BWZ and BLZ present a common dependence on the latent variable.

Following the confirmation of model specification, we performed power calculations for parameter estimation using the Shiny app *pwrSEM* [30]. Briefly, we calculated power to detect target effects with Monte Carlo simulations based on the specified MIMIC model, sample size, number and reliability of indicators of our latent variable, and the values of all other parameters in the model (coefficients, variances and covariances) [30]. We ran simulations in 1000 samples and derived power as the proportion of simulated samples that produced significant estimates of the regression coefficients in the measurement and structural models. The total study population was deemed sufficient to estimate all parameters with >0.80 power, except for the coefficient regarding the association of malaria during pregnancy and latent foetal growth conditions (powered at 0.70). The subsample was considered sufficient to estimate all coefficients related to the ultrasound biometric parameters with >0.80 power.

The inclusion of socioeconomic variables and stratification by a child’s sex in the sensitivity analyses did not affect estimates nor significantly increase the R^2^ of latent foetal growth conditions. We also performed analyses with a full information maximum likelihood estimator assuming missing at random. As estimates were virtually unchanged, we presented Satorra-Bentler adjusted estimates, for non-normality robustness. As evidence for cross-validation, histograms and 95% confidence intervals (95% CIs) of parameter values estimated from Monte Carlo methods using 1000 simulated samples for all coefficients in the measurement and structural models, considering the total study population and the subsample, are provided in the **Online Supplementary Document**. The interpretation of all associations was sustained in the simulations.

Finally, we obtained post-estimation linear predictions of latent foetal growth conditions after the MIMIC model. By fitting logistic regression models, we estimated odds ratios (ORs) and 95% CIs for the occurrence of preterm birth, reanimation procedures, infant formula prescription, and duration of hospital stay >3 days according to each standardised unit of predicted foetal growth conditions. As an alternative to these models, we additionally performed logistic regression models for the same set of perinatal variables according to each z-score of birth weight and length, separately, with adjustment for maternal age, height, pre-pregnancy weight, and elevated blood pressure, primiparity, smoking and malaria during pregnancy, and insufficient gestational weight gain.

We set statistical significance at *P* ≤ 0.05. We conducted all analyses in Stata, version 15 (StataCorp, College Station, Texas, USA).

## RESULTS

The participants had a median age of 24 years (IQR = 20, 30) and almost 40% had an inadequate pre-pregnancy nutritional status. We observed suboptimal antenatal care (<6 visits) among 22.3% of women, while 33.5% showed insufficient GWG (**Table 1**). Babies were born at a median 39.4 (IQR = 38.6, 40.0) gestational weeks. Mean BWZ and BLZ were compatible with the expected average birth size by international standards; 14.3% and 12.9% of newborns had BWZ and BLZ<-1, respectively. The subsample with ultrasound data presented better-off socioeconomic characteristics. Measured at a median 27.6 (IQR = 26.9, 28.6) gestational weeks, around 15% of foetuses had ultrasound biometric parameters<-1 z-score (**Table 2**).

**Table 1:** Characteristics of participants in the MINA-Brazil Study: total study population and subsample with assessments since the antenatal period

	Total (n = 1253)*	Subsample (n = 499)*
**Age in years**		
<19	235 (18.8)	99 (19.8)
19-34	899 (71.7)	353 (70.8)
≥35	119 (9.5)	47 (9.4)
**Skin colour**		
White	148 (11.8)	69 (13.8)
Non-white	1105 (88.2)	430 (86.2)
**Household wealth index†**		
1^st^ tercile –lowest	388 (31.0)	126 (25.6)
2^nd^ tercile	429 (34.2)	174 (34.9)
3^rd^ tercile –highest	436 (34.8)	199 (39.9)
**Schooling >9 y†**	762 (60.9)	349 (69.9)
**Assistance from the *Bolsa Família* Program**	529 (42.2)	199 (39.9)
**Living with a partner**	988 (78.9)	387 (77.6)
**Height in metres, median (IQR)**	1.57 (1.53, 1.61)	1.57 (1.53, 1.61)
**Pre-pregnancy weight in kilograms, median (IQR)**	57.3 (51.0, 65.0)	57.0 (51.0, 65.0)
**Pre-pregnancy nutritional status‡**		
Underweight	93 (7.4)	41 (8.2)
Normal weight	730 (58.3)	297 (59.5)
Overweight	311 (24.8)	119 (23.9)
Obesity	119 (9.5)	42 (8.4)
**Pre-pregnancy elevated blood pressure**	158 (12.6)	70 (14.0)
**Pre-pregnancy diabetes**	21 (1.7)	12 (2.4)
**Pre-pregnancy malaria†**	752 (60.0)	278 (55.7)
**Primiparity†**	498 (39.7)	226 (45.3)
**Planned pregnancy**	519 (41.4)	220 (44.1)
**Adequate antenatal care (≥6 visits)†**	964 (77.7)	416 (85.4)
**Supplement use during pregnancy**	488 (39.4)	195 (40.0)
**Smoking during pregnancy**	65 (5.2)	23 (4.6)
**Malaria during pregnancy**	94 (7.5)	35 (7.0)
**Insufficient gestational weight gain§**	418 (33.5)	154 (31.1)
**Child’s sex**		
Female	631 (50.4)	241 (48.3)
Male	622 (49.6)	258 (51.7)
**Birth weight in kilograms, median (IQR)**	3.26 (2.96, 3.56)	3.24 (2.95, 3.56)
**Birth weight for gestational age as z-score, median (IQR)**†**‖**	0.08 (-0.54, 0.79)	-0.02 (-0.60, 0.66)
**Birth length in centimetres, median (IQR)**	49.0 (48.0, 50.0)	49.0 (48.0, 51.0)
**Birth length for gestational age as z-score, median (IQR)†**	0.05 (-0.58, 0.82)	0.00 (-0.63, 0.70)
**Preterm birth¶**	94 (7.5)	39 (7.8)
**Reanimation procedures after birth†**	54 (4.3)	14 (2.8)
**Infant formula prescription**	161 (12.8)	64 (12.8)
**Duration of hospital stay >3 d**	81 (6.5)	33 (6.7)

IQR – interquantile range

*Data presented as n (%) unless otherwise specified. Totals may vary due to missing observations. Categorical and continuous variables were compared according to participation in assessments since the antenatal period using χ^2^ tests and Mann-Whitney tests, respectively.

† Significant differences (*P* < 0.05).

‡Categorised by body mass index (BMI) as underweight (<18.5 kg/m^2^), normal weight (≥18.5 to <25.0 kg/m^2^), overweight (≥25.0 to <30.0 kg/m^2^), and obesity (≥30.0 kg/m^2^) for adult women, and BMI-for-age z-score as underweight (<-2), normal weight (≥-2 to≤+1), overweight (>+1 to≤+2), and obesity (>+2) for adolescents, as defined by the World Health Organization.

§According to references from the Institute of Medicine.

‖According to the INTERGROWTH 21^st^ Project international standards for gestational age and sex.

¶According to gestational age at birth <37 weeks.

**Table 2:** Foetal biometric parameters of participants in the MINA-Brazil Study with ultrasound data during the antenatal period (n = 417)

	Median (IQR)*
**Gestational age in weeks**	27.6 (26.9, 28.6)
**Head circumference in millimetres**	255.0 (244.0, 267.0)
**Head circumference for gestational age as z-score†**	-0.25 (-0.84, 0.45)
**Small head circumference (<-1 z-score), n (%)†**	63 (15.1)
**Abdominal circumference in millimetres**	233.0 (219.0, 246.0)
**Abdominal circumference for gestational age (z-score)†**	0.22 (-0.45, 0.96)
**Small abdominal circumference (<-1 z-score), n (%)†**	59 (14.2)
**Femur length in millimetres**	51.0 (48.2, 53.5)
**Femur length for gestational age as z-score†**	0.03 (-0.62, 0.76)
**Small femur length (<-1 z-score), n (%)†**	59 (14.2)

IQR – interquartile range

*Values median and IQR unless otherwise specified.

†According to the INTERGROWTH 21^st^ Project international standards for gestational age.

Latent foetal growth conditions in the total population explained 88.3% of BWZ and 53.7% of BLZ variation (**Table 3**). The latent variable varied from 1.46 to 4.65, with a median value of 2.94 (IQR = 2.67, 3.21). Foetal growth conditions were continuously and positively associated with maternal height and pre-pregnancy weight, and inversely related to pre-pregnancy elevated blood pressure, primiparity, smoking, malaria episodes during pregnancy, and insufficient GWG, explaining 18.5% of the variation of the latent variable. Despite being observed in around 5.0% of participants, smoking during pregnancy showed the highest negative impact on foetal growth conditions (beta coefficient (β) = -0.55; 95% CI = -0.78, -0.32).

**Table 3:** Estimates for latent foetal growth conditions in the total study population and the subsample with ultrasound data in the MINA-Brazil study

	Total (n = 1249)*	Subsample (n = 400)*
**Structural model†**		
Maternal characteristics		
*Age in years*	-0.00 (-0.01, 0.01)	0.01 (-0.01, 0.02)
*Height in centimetres*	0.01 (0.01, 0.02)	0.02 (0.01, 0.04)
*Pre-pregnancy weight in kilograms*	0.02 (0.01, 0.02)	0.01 (0.01, 0.02)
*Pre-pregnancy elevated blood pressure*	-0.22 (-0.40, -0.05)	-0.42 (-0.62, -0.22)
*Primiparity*	-0.29 (-0.41, -0.18)	-0.19 (-0.37, -0.01)
*Smoking during pregnancy*	-0.55 (-0.78, -0.32)	-0.58 (-0.95, -0.21)
*Malaria during pregnancy*	-0.17 (-0.31, -0.04)	-0.04 (-0.28, 0.19)
*Insufficient gestational weight gain*	-0.36 (-0.46, -0.25)	-0.32 (-0.50, -0.14)
Foetal biometry for gestational age‡		
*Head circumference as z-score*	-	0.18 (0.10, 0.26)
*Abdominal circumference as z-score*	-	0.28 (0.20, 0.36)
*Femur length as z-score*	-	0.11 (0.04, 0.19)
**Measurement model§**		
Size at birth		
*Birth weight as z-score*	1, R^2^ = 0.88	1, R^2^ = 0.81
*Birth length as z-score*	0.82 (0.71, 0.93), R^2^ = 0.54	0.83 (0.70, 0.97), R^2^ = 0.52
**R^2^ for latent foetal growth conditions**	0.19	0.44
**SRMR‖**	0.02	0.02

β – beta coefficient, CI – confidence interval. SRMR – standardised root mean squared residual

*Data presented as β (95% CI) unless otherwise specified. We estimated all models using maximum likelihood with Satorra-Bentler scaled χ^2^ statistic and corresponding robust standard errors.

†The structural model for latent foetal growth conditions among the total study population included maternal age, height, pre-pregnancy weight, pre-pregnancy elevated blood pressure, primiparity, smoking during pregnancy, malaria during pregnancy, and insufficient gestational weight gain. Additional paths were indicated from maternal height and pre-pregnancy weight to pre-pregnancy elevated blood pressure; and from maternal pre-pregnancy weight, primiparity, and smoking during pregnancy to insufficient gestational weight gain. For the subsample with ultrasound data during the antenatal period, head circumference, abdominal circumference, and femur length z-scores for gestational age were additionally considered.

‡According to the INTERGROWTH 21^st^ Project international standards for gestational age.

§The measurement model for latent foetal growth conditions included birth weight and length z-scores, calculated according to the INTERGROWTH 21^st^ Project international standards for gestational age and sex.

‖SRMR indicated the average difference between the observed correlation and the model implied correlation matrix (ideally <0.08).

The direction and significance of all associations were sustained in the subsample analysis, except for the occurrence of malaria. HC, AC, and FL assessed at the beginning of the third trimester were positively associated with foetal growth conditions, with the R^2^ markedly increasing to 43.8%. Foetal AC (β = 0.28; 95% CI = 0.20, 0.36) had a relatively larger expression in the latent variable than FL (β = 0.11; 95% CI = 0.04, 0.19).

**Figure 2:**
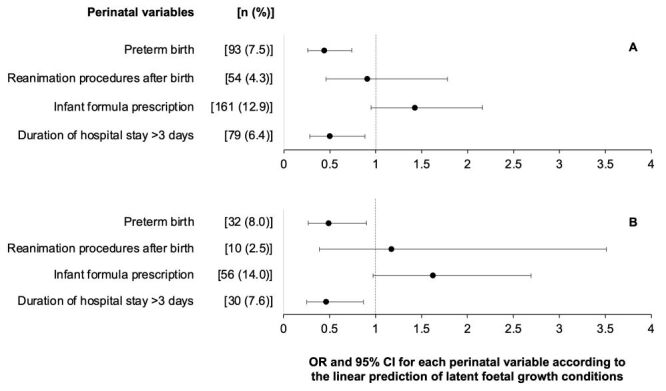
Association of the linear prediction of latent foetal growth conditions with perinatal variables in the MINA-Brazil Study, following models in **Figure 1**. Odds ratios (OR) and 95% confidence intervals (95% CI) were estimated using logistic regression models. **Panel A.** Estimates for the total study population. **Panel B.** Estimates for the subsample with ultrasound data.

Each standardised unit of predicted foetal growth conditions halved the chance for the occurrence of preterm birth (95% CI = 0.26, 0.74) and hospital stay >3 days (95% CI = 0.28, 0.88) (**Figure 2**). We observed no association between reanimation procedures after birth and infant formula prescription. We had similar findings for the subsample with ultrasound data. Conversely, BWZ and BLZ were not independently associated with these perinatal variables in separate logistic regression models (Figure S3 in the **Online Supplementary Document**).

## DISCUSSION

We confirmed the initial hypotheses for this study. The prediction of foetal growth conditions improved with information on maternal morbidities, primiparity, smoking during pregnancy, insufficient GWG, and ultrasound biometric parameters, accounting for up to 40% of total variation in the latent variable. We also found that better foetal growth conditions were associated with a lower occurrence of preterm birth and a shorter hospital stay after delivery.

This analysis expanded the understanding of foetal growth conditions through interpretative and analytical perspectives in relation to previous investigations [11,12]. Instead of relying on crude birth size, we adopted a prescriptive interpretation for the latent variable by using z-scores from international standards for optimal growth patterns [13,15] as indicator variables. The rationale for cohort selection in the construction of the INTERGROWTH-21^st^ Project charts [31] was reflected in adequate postnatal growth, satisfactory gross motor development, and continuously low morbidity rates up to two years of age [32]. Analytically, we identified a larger set of independent determinants and explained a much greater proportion of variation in latent foetal growth conditions in comparison to earlier models [11,12], more adequately informing a metric to quantify the underlying environment for intrauterine development.

Pre-pregnancy maternal anthropometry favoured foetal growth, while primiparity and smoking were negatively associated with latent conditions connecting the distinct components of newborn size. Demonstrating intergenerational influences on the nutritional status of populations, a recent analysis with pooled data from 65 061 children in 20 cohorts in low- and middle-income countries (LMICs) concatenated positive associations of maternal height and BMI on child’s linear growth and weight gain from birth to two years of age [33]. Population intervention effects for shifting birth order to firstborn incurred in a -0.14 z-score lower length (95% CI = -0.24, -0.04) and a -0.39 z-score lower weight for length (95% CI = -0.76, -0.02) at birth [33], similar to our estimates in direction and magnitude. Detrimental impacts of smoking during pregnancy were found to double the chance for low birth weight (95% CI = 1.77, 2.26) in meta-analysed data from the Americas [34], in agreement with our results.

We found independent effects of elevated blood pressure and malaria infection on latent foetal growth conditions. A previous meta-analysis observed greater odds for adverse outcomes, including small-for-gestational-age newborns (OR = 1.96; 95% CI = 1.60, 2.40) for high blood pressure predating pregnancy [35]; lower attained weight and length z-scores have been reported up to age 36 months [36]. There is concerning evidence of accelerating upward trends of pre-pregnancy hypertension in all age groups during the past decade, with a higher burden indicated in rural, less urbanised areas [37]. Concurrently, clinical impacts of *Plasmodium vivax* malaria have been documented among pregnant women and their infants [38]. A systematic review estimated a 63% higher risk for low birth weight (95% CI = 1.48, 1.80) due to exposure to pregnancy-associated malaria [39]. Randomised trials demonstrated that broader infection control measures resulted in improvements in maternal nutritional status and birth size [40], and lower stunting incidence and better developmental scores at five years of age [41]. These results underscore cumulative disease burdens whose consequences are closely connected to limited access to health care before and during pregnancy, primarily affecting populations in LMICs.

We observed an important contribution of ultrasound biometric parameters to latent foetal growth conditions in our subsample analysis, with a relatively larger association with AC. Examination of HC, AC, and FL enables a comprehensive assessment of structures with distinct growth patterns [42] and repercussions on neonatal body composition [43]. Development of skeletal parameters was found to be concentrated in early pregnancy, especially FL. Conversely, AC accrual had a steadier rate until term [42], encompassing the progress for most vital organs, crucial for foetal growth conditions, alongside subcutaneous fat. Third-trimester scans may enhance the detection of smallness for GA and neonatal morbidity, particularly when decreased AC growth velocity is observed [44], which is consistent with our study.

Our finding that predicted foetal growth conditions were related to reduced odds for preterm birth and longer hospital stays is in line with updated analyses of causes of child mortality. In 2019, preterm birth complications (16.6%) and intrapartum-related events (11.0%) accounted for the highest fractions of neonatal deaths globally [45]. Furthermore, as a proof-of-principle, these results were reassuring of the approach to latent conditions connecting BWZ and BLZ (whose proportions of explained variation were high, at 88.3% and 53.7%, respectively). While existing studies are mostly dedicated to birth weight, modelling separate birth size components could not acknowledge significant associations with perinatal variables in our analysis. This key finding suggests that the latent variable may capture more than intrauterine weight or fat accrual, including length gain. This is an important distinction, with potential implications for future health and well-being of populations. For instance, pooled estimates from longitudinal studies recently highlighted that birth length projected at >50 cm was the most impactful factor for improvements in the nutritional status of children at two years of age [33]. Improved linear growth has been reliably linked to increased education attainment and higher lifetime earnings, with repercussions for analyses of relative costs and benefits of the implementation of nutritional interventions in LMICs [46].

Overall, the results from the structural model of latent foetal growth conditions and the estimates regarding its influence on perinatal variables point to a renewed attention to determinants of conditions that jointly engender BWZ and BLZ in epidemiological studies. Such conditions may warrant stronger implications for the promotion of better intrauterine environment, perinatal health, and child growth in its various dimensions. Future research is needed to translate this work into clinical settings and policymaking processes. Further studies may clarify whether latent foetal growth conditions relate to the risk of metabolic or cardiovascular disease in later years, and if incorporating such a metric could improve current cost-effective and benefit-cost estimates to prioritise the use of financial resources in promoting human capital and mitigating adversities since gestation.

This study has some limitations. Self-reported data and information retrieved from medical records are subject to measurement bias, even though standardised training was provided for health professionals in the local maternity hospital. Agreement of such measures with a set of procedures conducted directly by the study team has also been assessed and considered satisfactory. Findings related to the exposure to malaria during pregnancy (affecting 7.5% of the study population) should be interpreted with caution, as our model had a somewhat lower statistical power to detect this association. Additionally, estimates may not extrapolate to settings where malaria infection is not a public health concern. Logistic limitations prevented follow-up during pregnancy of participants in remote rural areas. Socioeconomic differences in the subsample with ultrasound data were therefore expected, but may affect the generalisability of estimates, as magnitude of associations could have been larger in a more heterogeneous population. Nevertheless, the consistency across overall and subsample findings was expressive and should be noted amid the robustness of this analysis. Among several strengths of this study are high-quality ultrasound data collected in a low-resource setting, besides population-based information available on birth outcomes and perinatal variables. Through the analytical framework employed here, the distinct newborn size components of weight and length could be statistically instrumentalised in an integrated approach to foetal growth conditions that may aid the identification of pivotal determinants in epidemiological analysis in global health.

## CONCLUSIONS

In a scenario of dual burden of maternal morbidities, primiparity, elevated blood pressure, smoking, and malaria infection negatively influenced latent foetal growth conditions. Encompassing both weight gain and linear growth during gestation, these conditions had perinatal implications for preterm birth and duration of hospital stay after delivery. Such metric for foetal growth conditions may aid strategies favouring an integrated approach to child growth in a sensitive window of opportunity for health promotion.

## Additional material


Online Supplementary Document
