# Supplementary material for: Improved estimates of foetal growth are associated with perinatal outcomes: A latent modelling approach in a population-based birth cohort
Source: J Glob Health. 2023 Sep 11;13:04070. doi: 10.7189/jogh.13.04070 (PMC10494278; doi:10.7189/jogh.13.04070)
Supplement: Online Supplementary Document [file jogh-13-04070-s001.pdf]

Improved estimates of foetal growth are associated with perinatal outcomes: A latent modelling approach in a population-based birth cohort

Bárbara H. Lourenço, Paulo A. R. Neves, Marly A. Cardoso, Marcia C. Castro; on behalf of the MINA-Brazil Study Group

Online Supplementary Document

- Figure S1: Flowchart of participants in the MINA-Brazil Study included in the analysis.
- Figure S2: Model representation for direct effects of maternal characteristics and ultrasound biometric parameters on size at birth in the MINA-Brazil Study.
- Table S1: Goodness-of-fit estimates for model selection.
- Figure S3: Associations of birth weight and length z-scores for gestational age with perinatal variables in the MINA-Brazil Study.
- Figures S4–S26: Set of histograms of parameter values and 95% confidence intervals (95% CI) estimated from Monte Carlo simulations in the total study population and in the subsample in the MINA-Brazil Study.

Figure S1. Flowchart of participants in the MINA-Brazil Study included in the analysis.

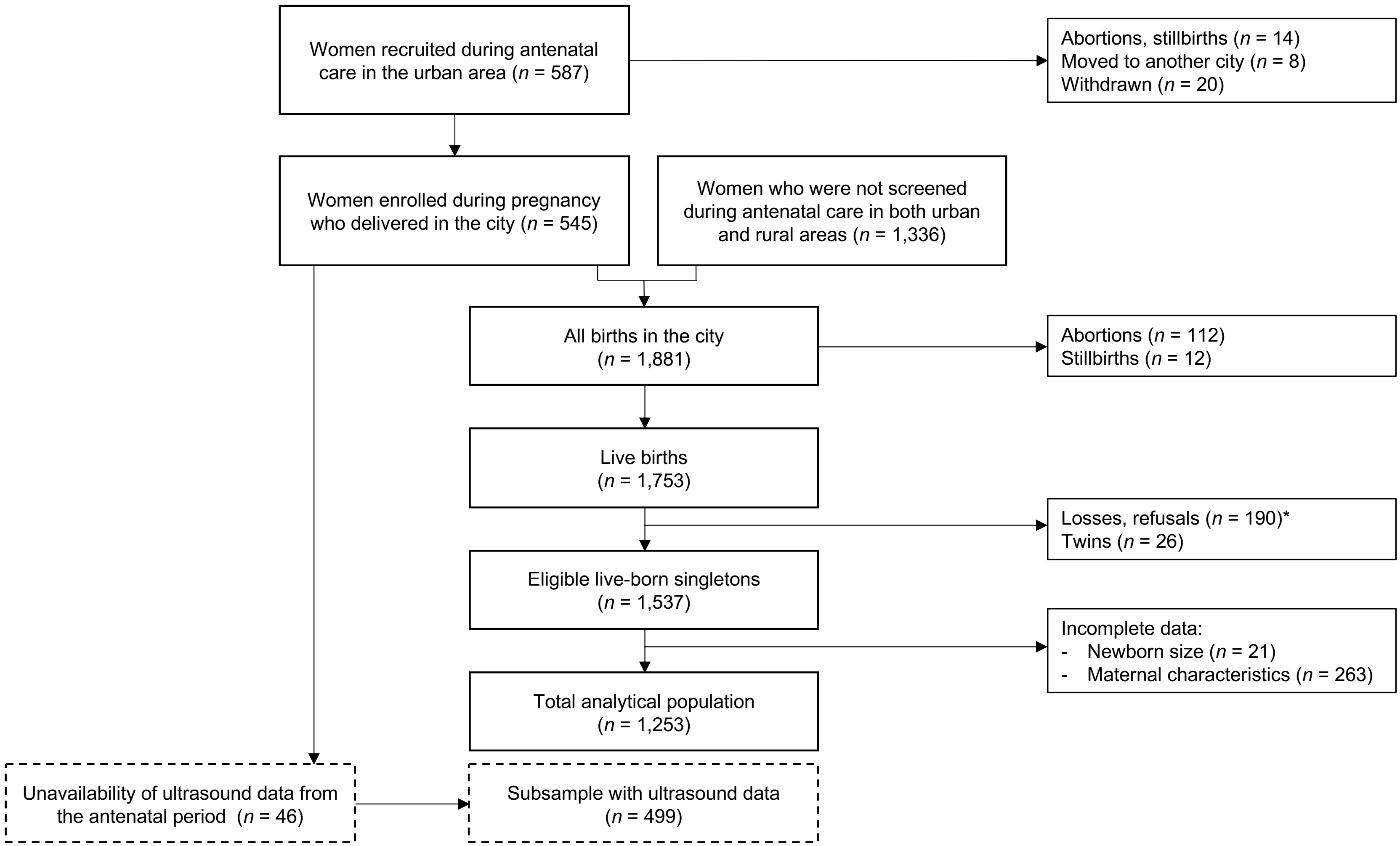

*Consolidated total losses and refusals after retrieving data from participation in the MINA-Brazil Study during the antenatal period.

Figure S2. Model representation for direct effects of maternal characteristics and ultrasound biometric parameters on size at birth in the MINA-Brazil Study.

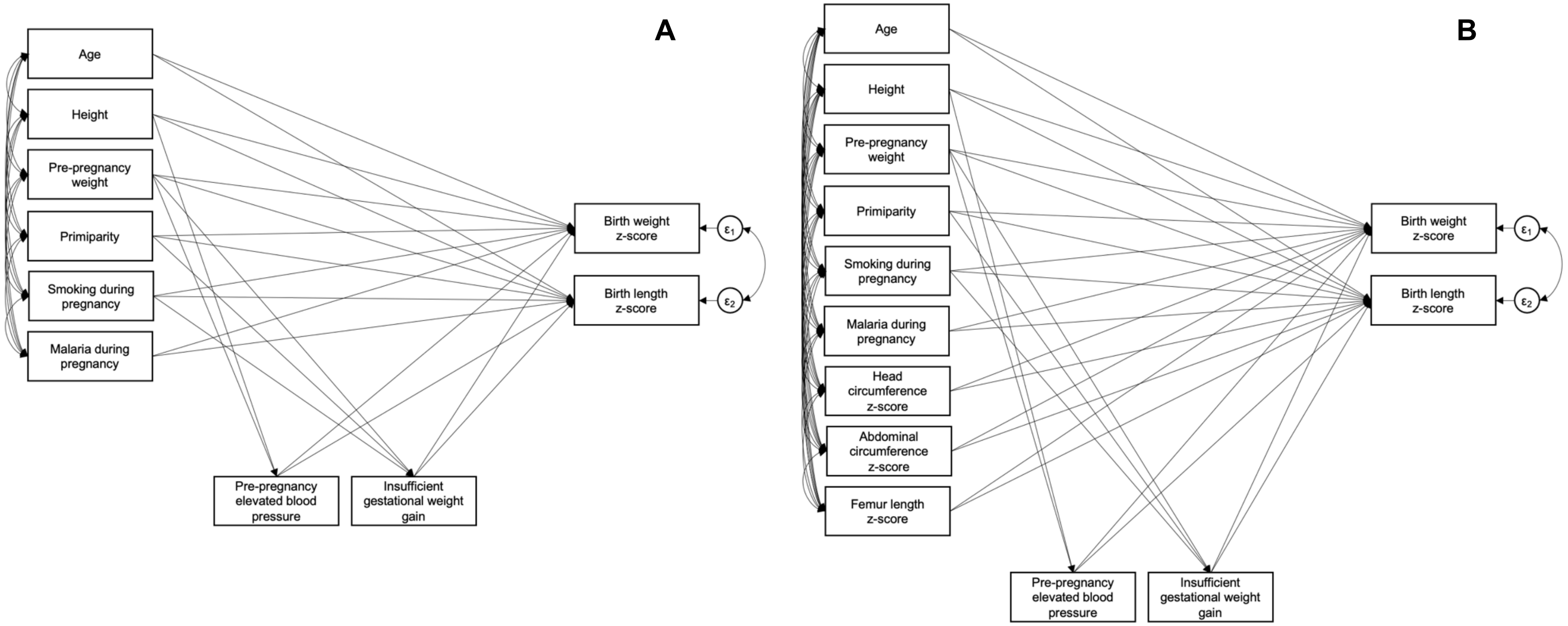

Panel A. Model representation for the total study population. **Panel B.** Model representation for the subsample with ultrasound data during the antenatal period.

Table S1. Global fit measures for structural equation models with direct effects or mediation with latent foetal growth conditions on size at birth in the MINA-Brazil Study

	Test statistic	df	*P*	RMSEA	BIC	CFI	TLI
**Total study population (n=1249)***							
Direct effects model	11.73	8	0.16	0.019	36809	0.996	0.986
Mediated effects model	17.11	15	0.31	0.011	36764	0.998	0.996
**Subsample (n=400)†**							
Direct effects model	8.47	14	0.86	0.000	15062	1.000	1.000
Mediated effects model	20.77	24	0.65	0.000	15014	1.000	1.000

RMSEA: root mean squared error of approximation. BIC: Bayesian information criterion. CFI: comparative fit index. TLI: Tucker-Lewis index. All models were estimated using maximum likelihood with Satorra-Bentler scaled χ^2 statistic and corresponding robust standard errors.

*For the total study population, in the direct effects models each maternal predictor had a direct effect on birth weight and length z-scores for gestational age and sex (see Figure S1). In mediated effects models, the effect of each maternal characteristic on size at birth was mediated by a latent variable for foetal growth conditions (see Figure 1). Maternal characteristics were age, height, pre-pregnancy weight, pre-pregnancy elevated blood pressure, primiparity, smoking during pregnancy, malaria during pregnancy, and insufficient gestational weight gain. Additional paths were indicated from maternal height and pre-pregnancy weight to pre-pregnancy elevated blood pressure; and from maternal pre-pregnancy weight, primiparity, and smoking during pregnancy to insufficient gestational weight gain.

†For the subsample with ultrasound data during the antenatal period, head circumference, abdominal circumference, and femur length z-scores for gestational age were additionally considered.

Figure S3. Associations of birth weight and length z-scores for gestational age with perinatal variables in the MINA-Brazil Study

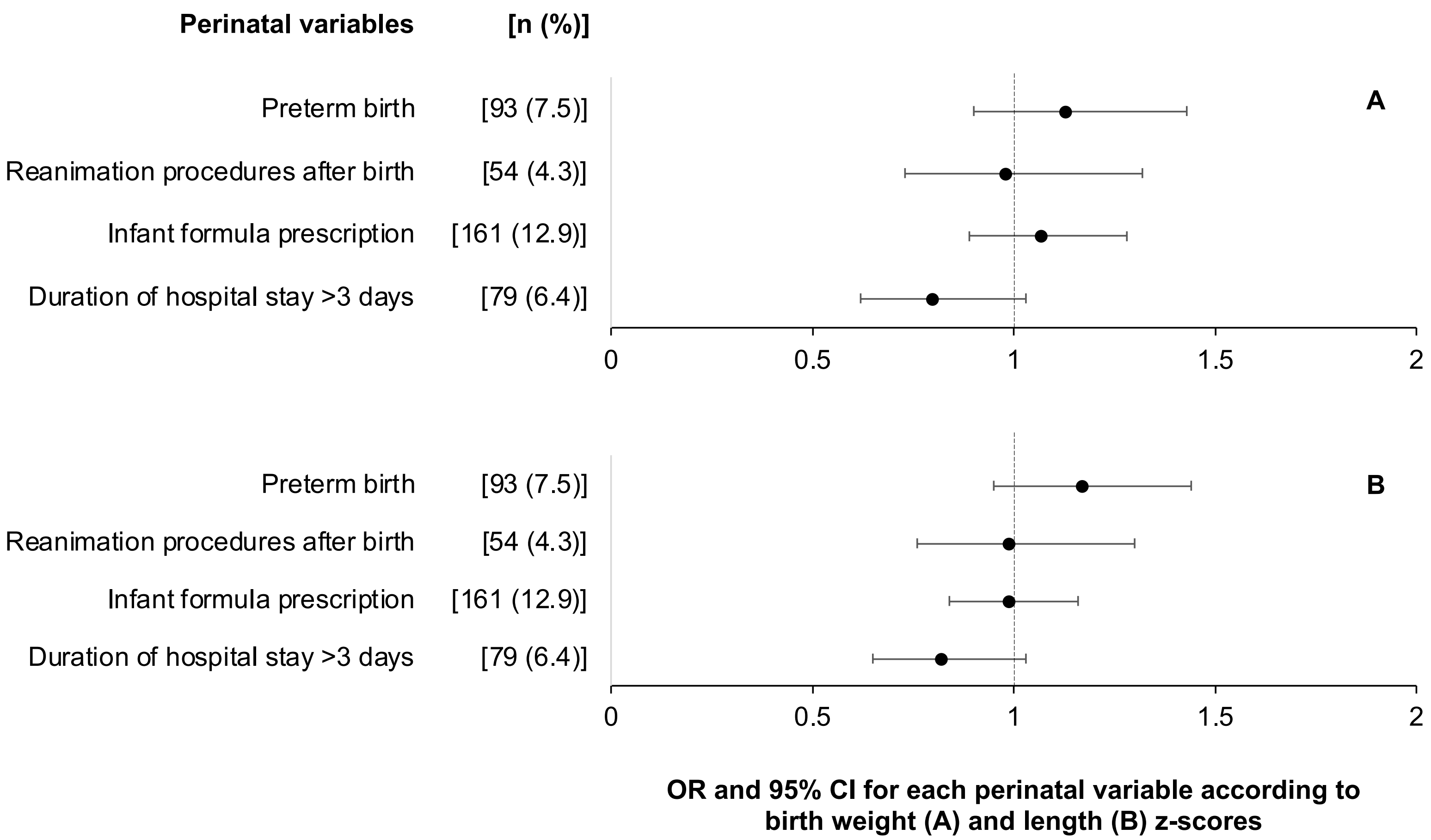

In the total study population, odds ratio (OR) and 95% confidence intervals (95% CI) were estimated for perinatal variables for each z-score of newborn size according to gestational age and sex, using logistic regression models with adjustment for maternal age, height, pre-pregnancy weight, and elevated blood pressure, primiparity, smoking and malaria during pregnancy, and insufficient gestational weight gain. **Panel A.** Estimates for birth weight z-score. **Panel B.** Estimates for birth length z-score.

Set of histograms of parameter values and 95% confidence intervals (95% CI) estimated from Monte Carlo methods using 1000 simulated samples. Simulations were based on the specified model for latent foetal growth conditions and sample size, at an alpha level of 0.05. Parameters of interest included regression coefficients in the measurement and structural models for the total study population and the subsample.

Analyses were run and histograms were generated in the Shiny app *pwrSEM*, which is freely available online at https://yilinandrewang.shinyapps.io/pwrSEM/. Vertical solid line indicates the population value set for the parameter; vertical dotted line indicates the median of parameter estimates from the simulated samples.

Model for the total study population (n=1249)

Figure S4. Estimated parameter values for the factor loading of birth weight z-score, measurement model in the total study population in the MINA-Brazil Study.

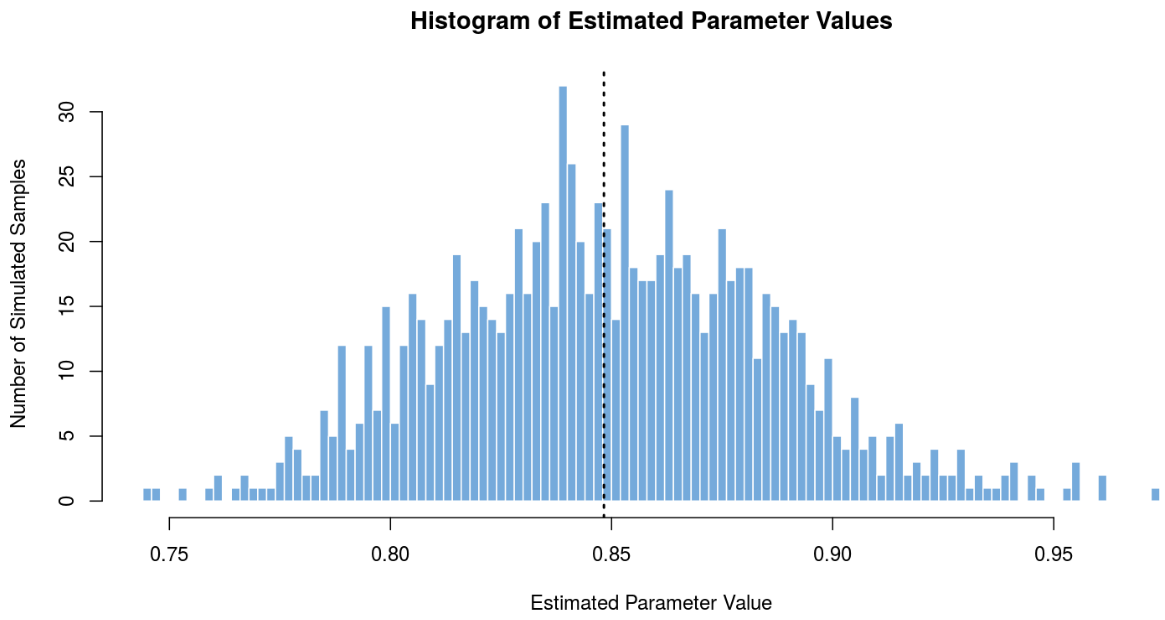

95% CI of estimates for birth weight z-score: 0.78, 0.93

Figure S5. Estimated parameter values for the factor loading of birth length z-score, measurement model in the total study population in the MINA-Brazil Study.

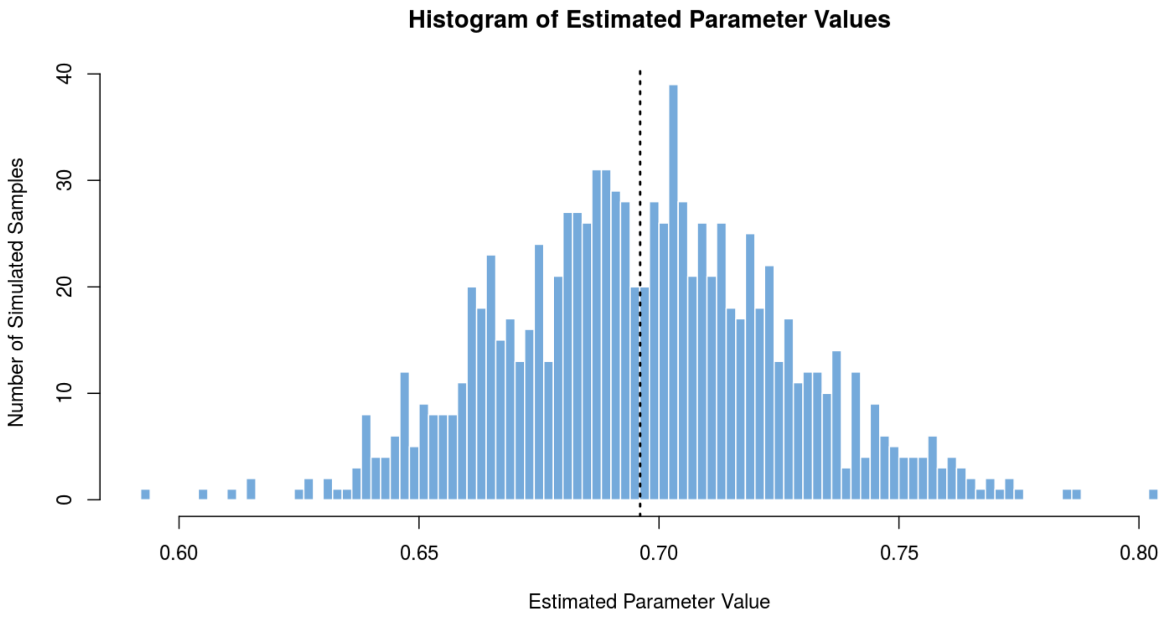

95% CI of estimates for birth length z-score: 0.64, 0.76

Figure S6. Estimated parameter values for the coefficient of age (years), structural model in the total study population in the MINA-Brazil Study.

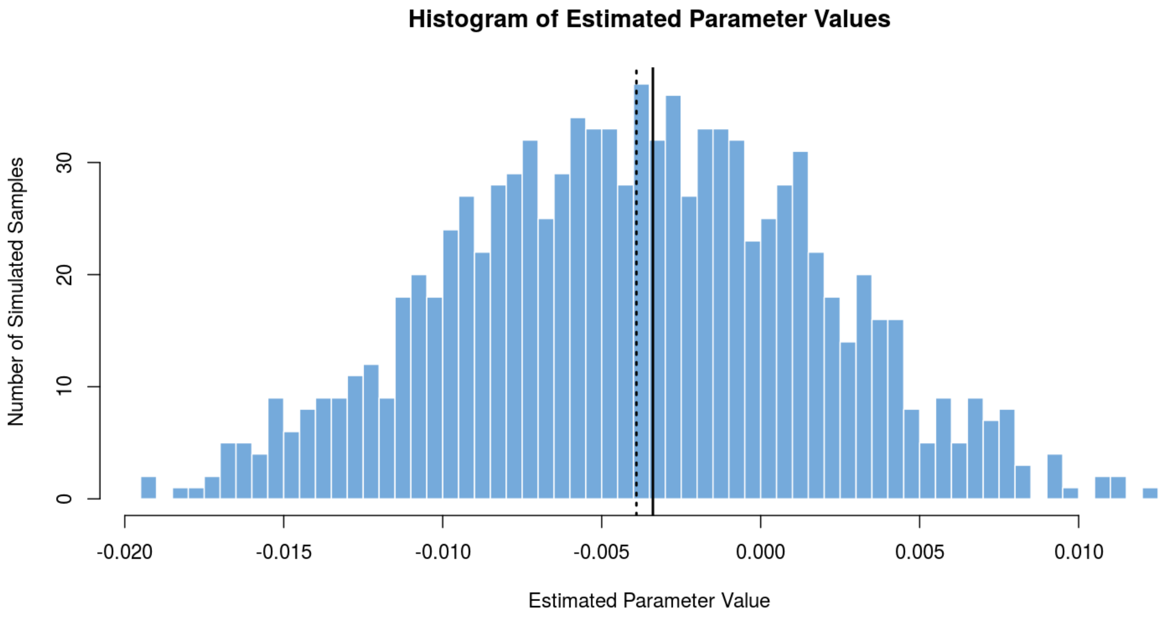

95% CI of estimates for age (years): -0.02, 0.01

Figure S7. Estimated parameter values for the coefficient of height (cm), structural model in the total study population in the MINA-Brazil Study.

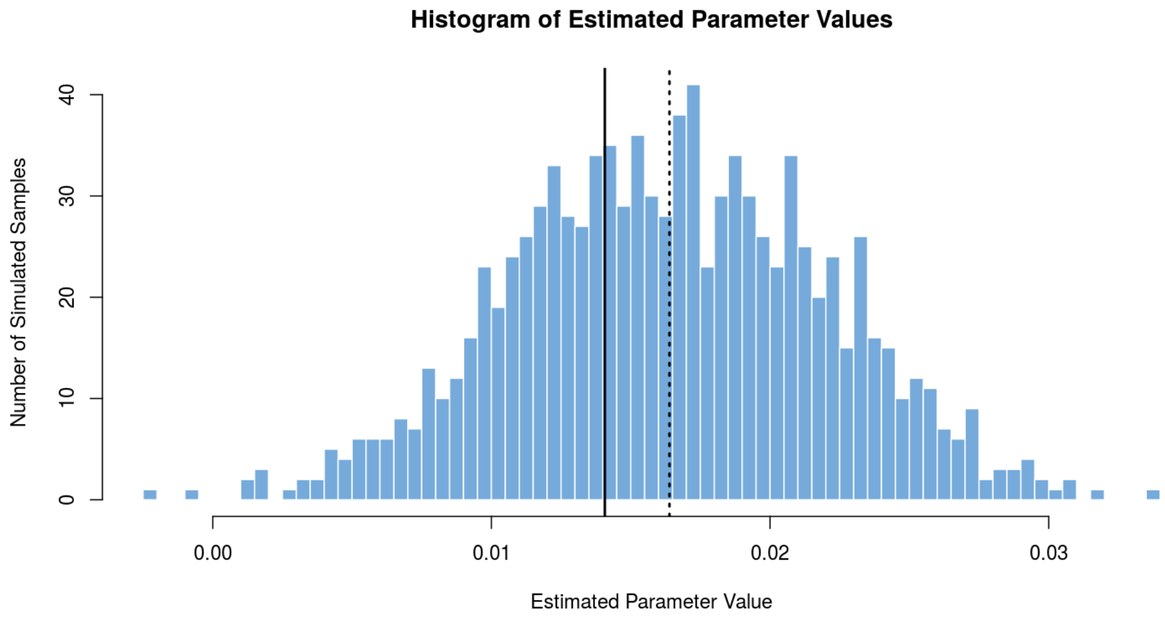

95% CI of estimates for height (cm): 0.01, 0.03

Figure S8. Estimated parameter values for the coefficient of pre-pregnancy weight (kg), structural model in the total study population in the MINA-Brazil Study.

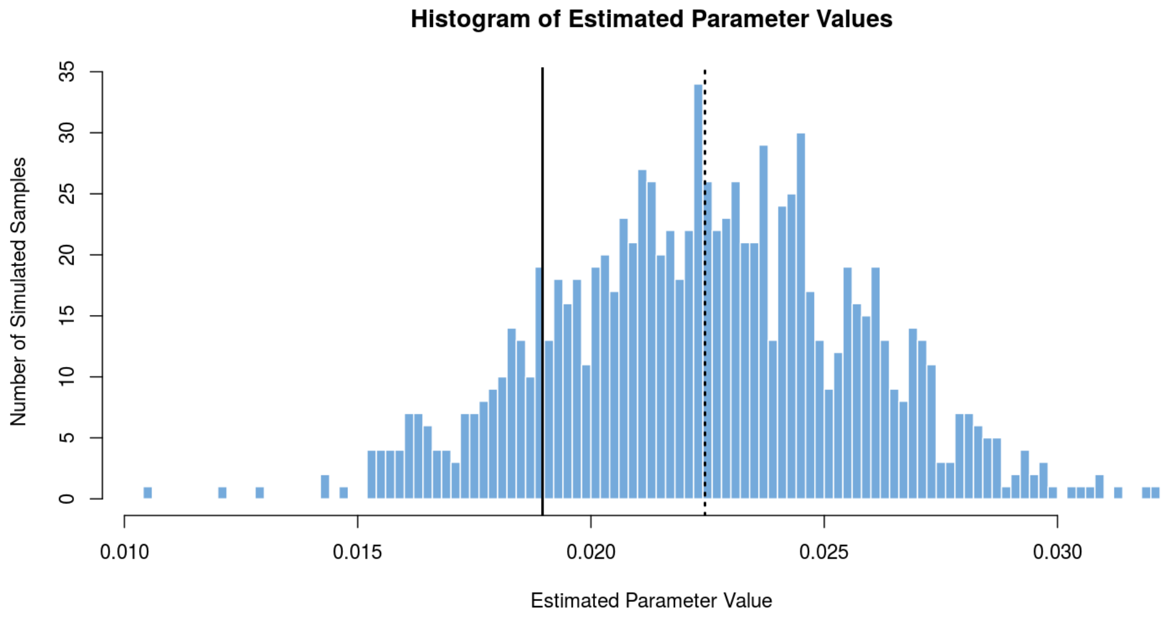

95% CI of estimates for pre-pregnancy weight (kg): 0.02, 0.03

Figure S9. Estimated parameter values for the coefficient of pre-pregnancy elevated blood pressure, structural model in the total study population in the MINA-Brazil Study.

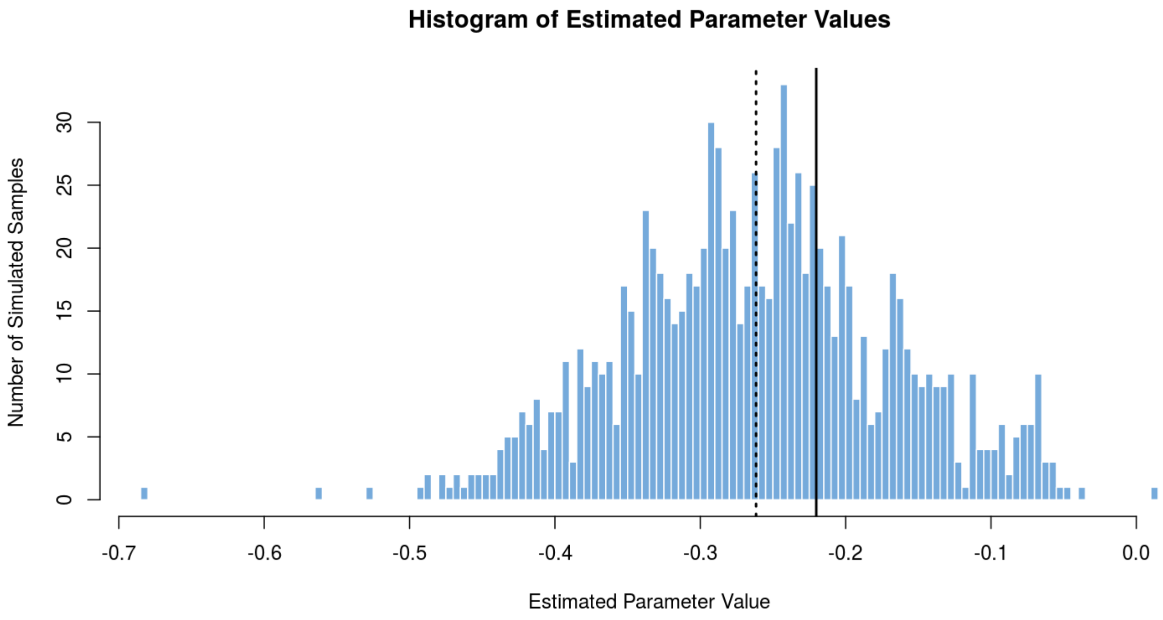

95% CI of estimates for pre-pregnancy elevated blood pressure: -0.43, -0.07

Figure S10. Estimated parameter values for the coefficient of primiparity, structural model in the total study population in the MINA-Brazil Study.

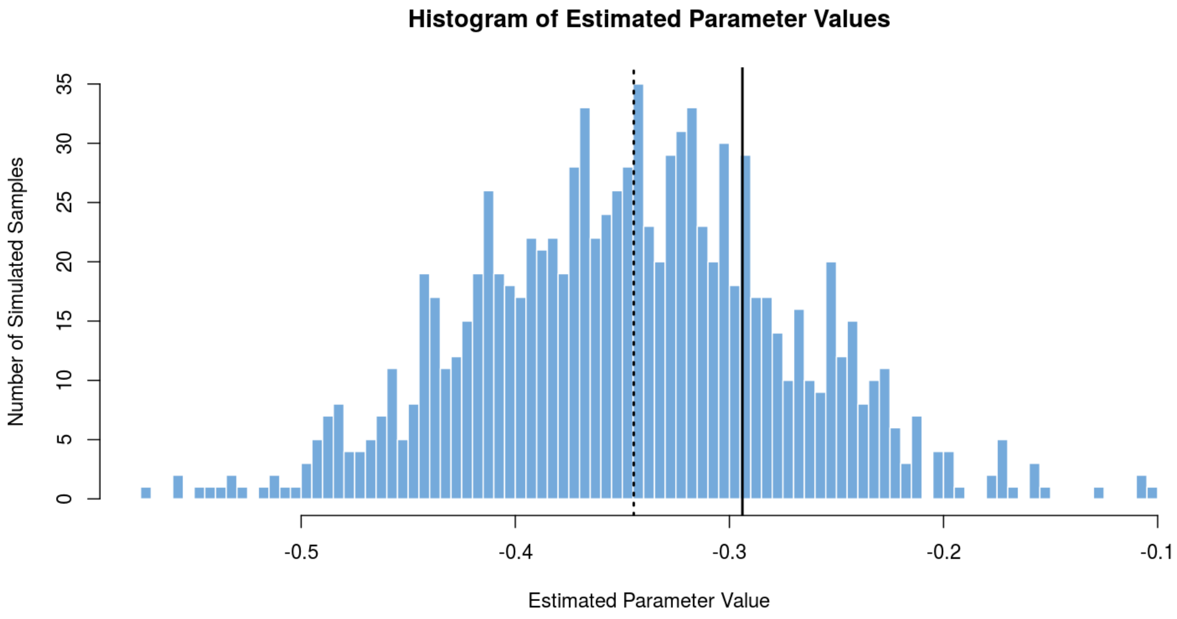

95% CI of estimates for primiparity: -0.49, -0.21

Figure S11. Estimated parameter values for the coefficient of smoking during pregnancy, structural model in the total study population in the MINA-Brazil Study.

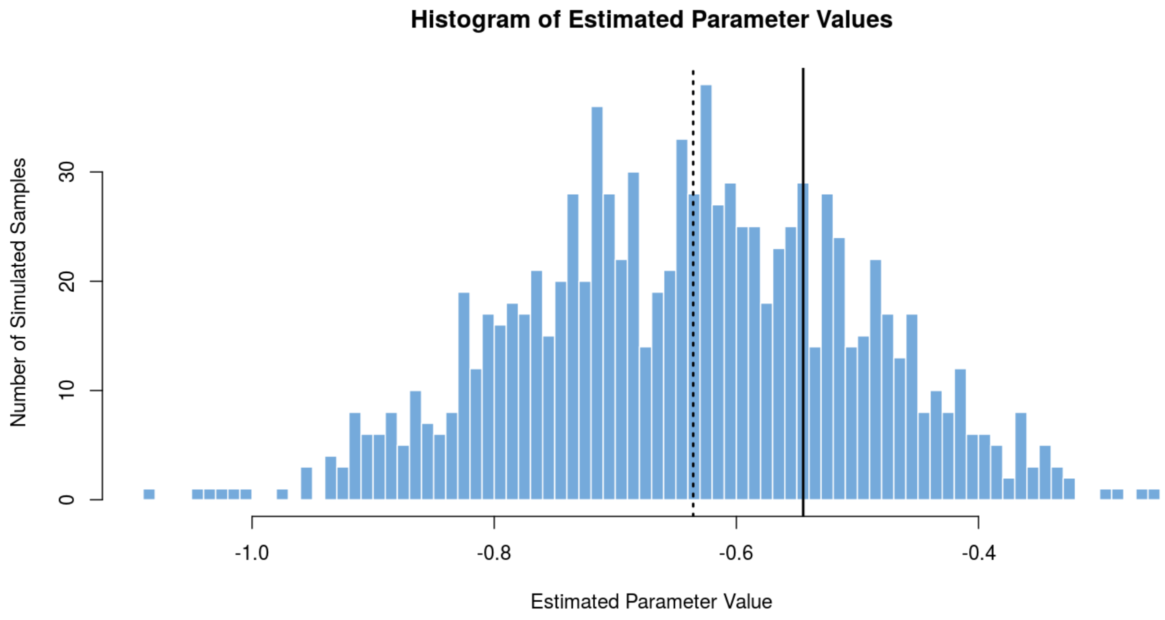

95% CI of estimates for smoking during pregnancy: -0.91, -0.37

Figure S12. Estimated parameter values for the coefficient of malaria during pregnancy, structural model in the total study population in the MINA-Brazil Study.

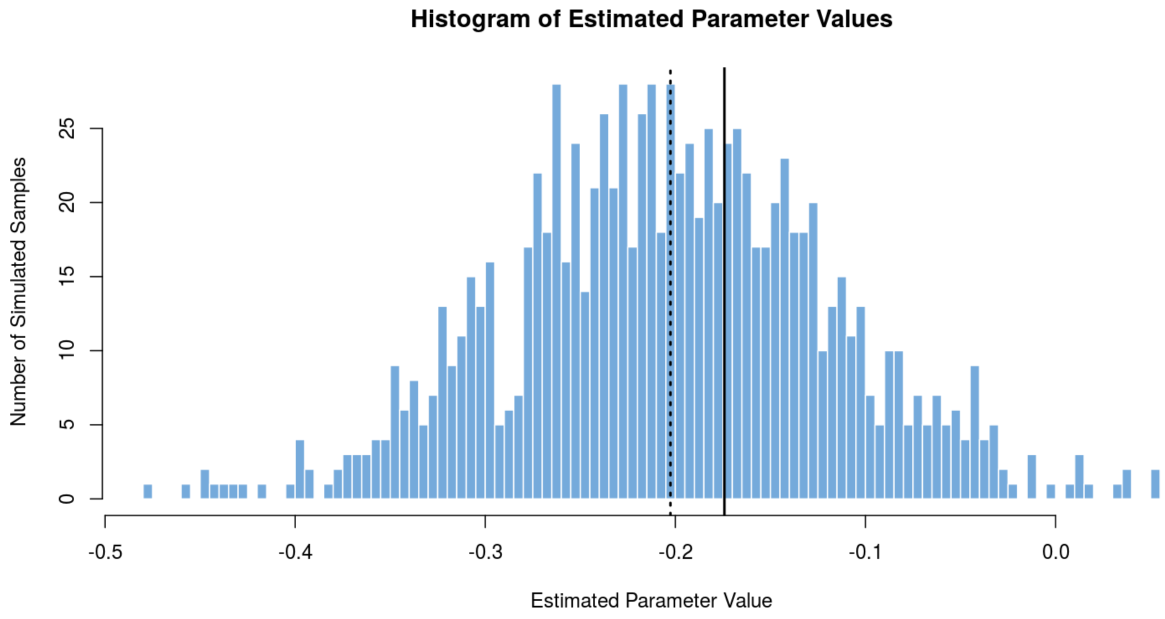

95% CI of estimates for malaria during pregnancy: -0.37, -0.04

Figure S13. Estimated parameter values for the coefficient of insufficient gestational weight gain, structural model in the total study population in the MINA-Brazil Study.

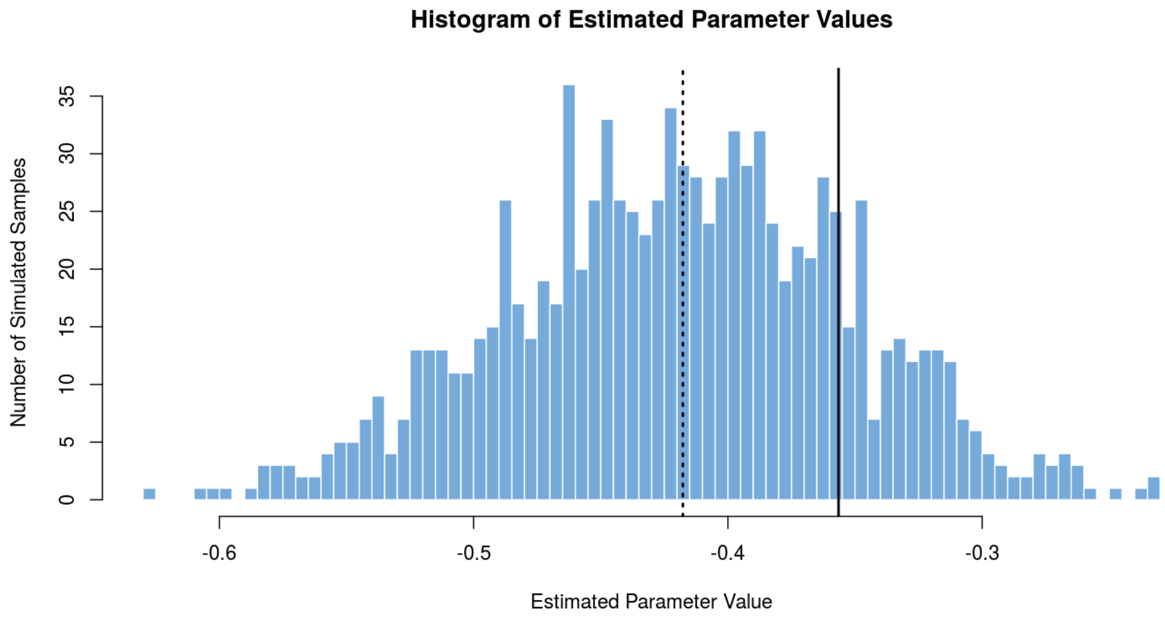

95% CI of estimates for insufficient gestational weight gain: -0.55, -0.29

Model for the subsample (n=400)

Figure S14. Estimated parameter values for the factor loading of birth weight z-score, measurement model in the subsample in the MINA-Brazil Study.

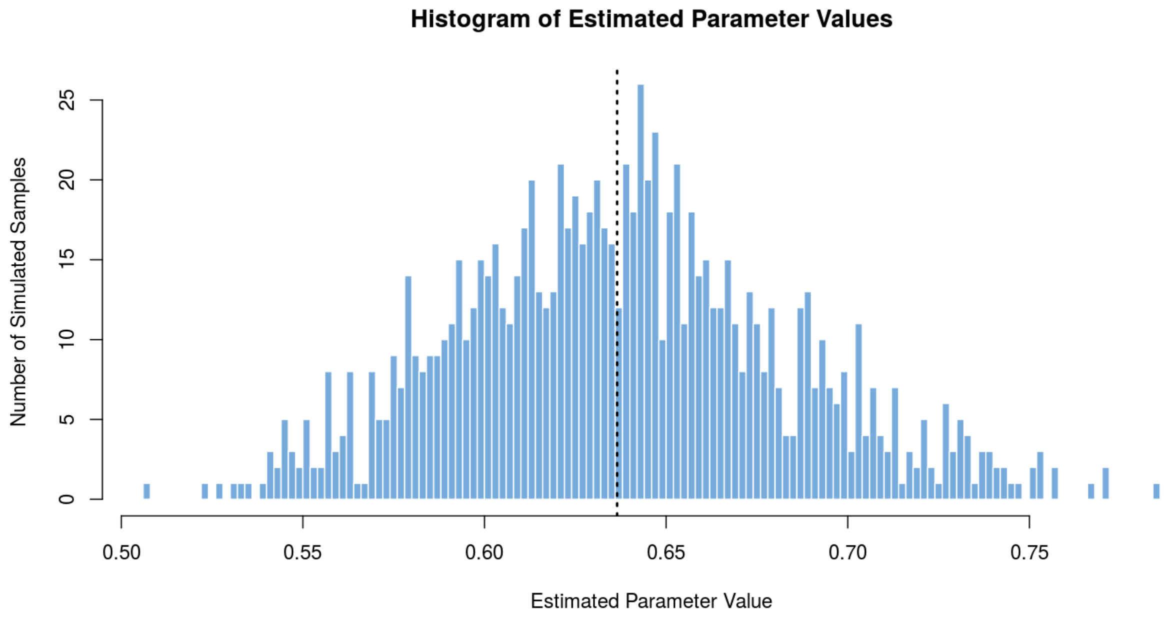

95% CI of estimates for birth weight z-score: 0.55, 0.73

Figure S15. Estimated parameter values for the factor loading of birth length z-score, measurement model in the subsample in the MINA-Brazil Study.

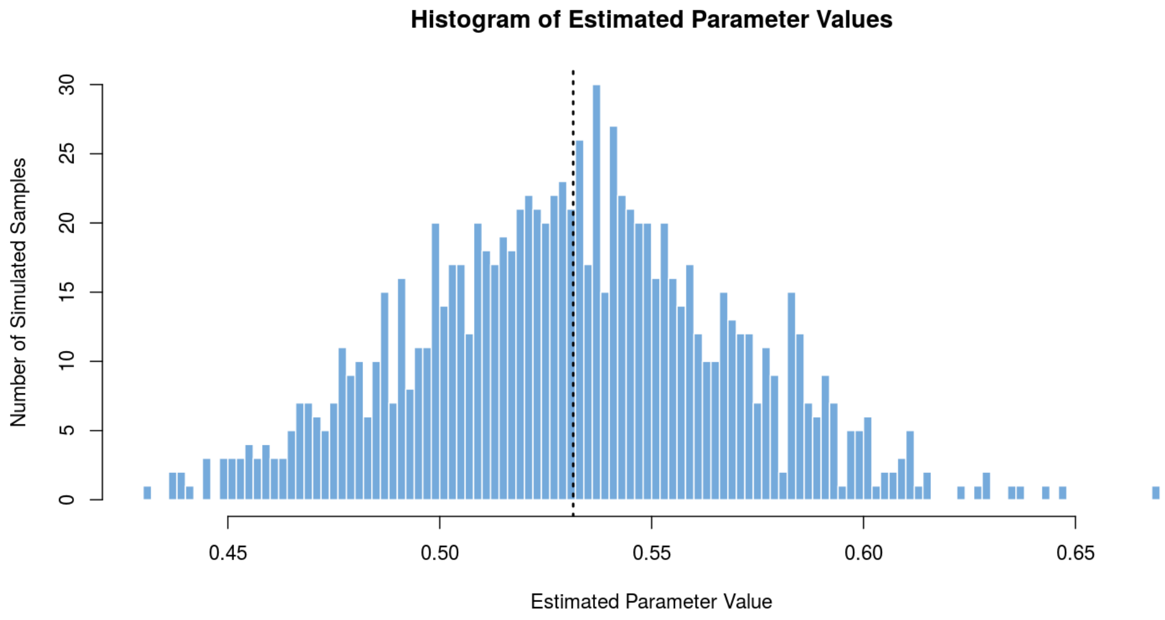

95% CI of estimates for birth length z-score: 0.46, 0.60

Figure S16. Estimated parameter values for the coefficient of age (years), structural model in the subsample in the MINA-Brazil Study.

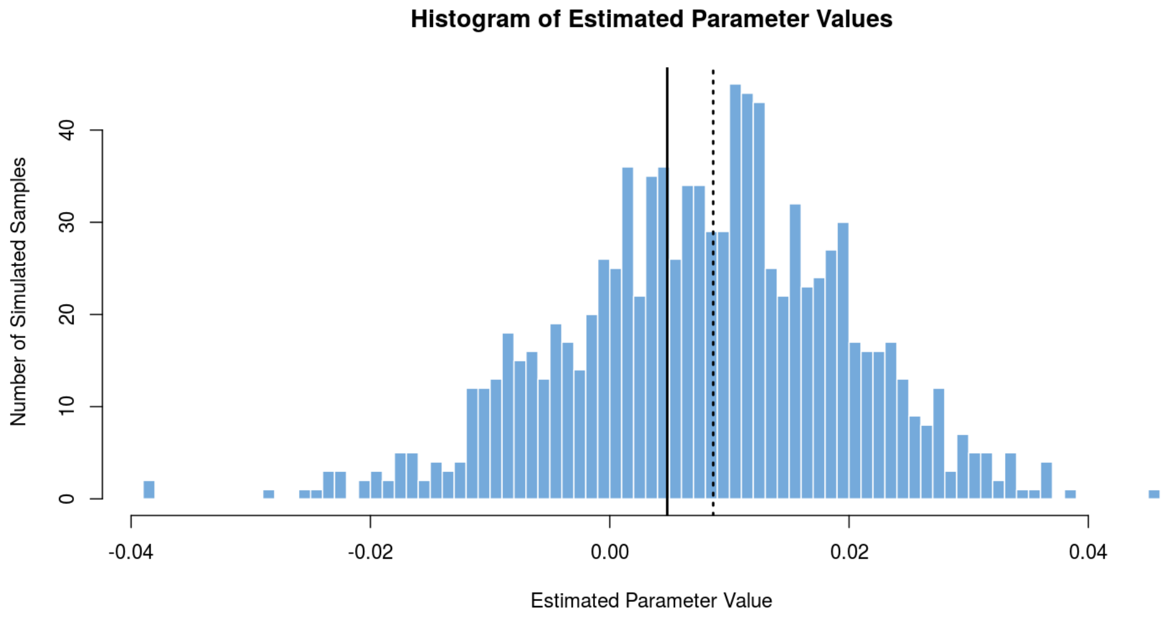

95% CI of estimates for age (years): -0.02, 0.03

Figure S17. Estimated parameter values for the coefficient of height (cm), structural model in the subsample in the MINA-Brazil Study.

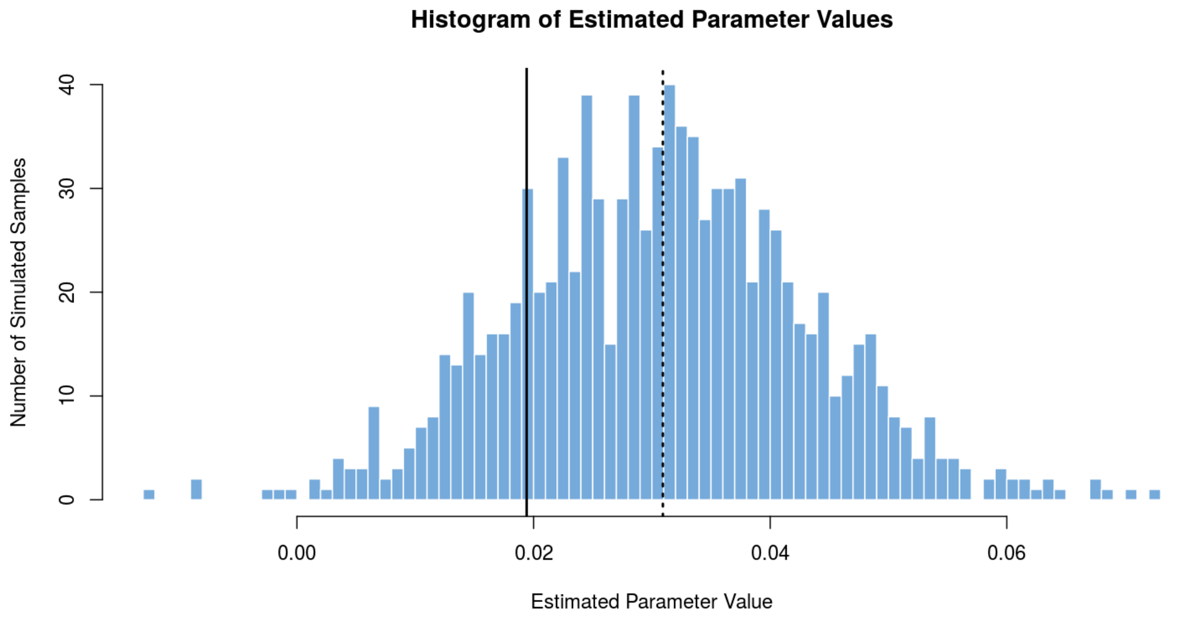

95% CI of estimates for height (cm): 0.01, 0.05

Figure S18. Estimated parameter values for the coefficient of pre-pregnancy weight (kg), structural model in the subsample in the MINA-Brazil Study.

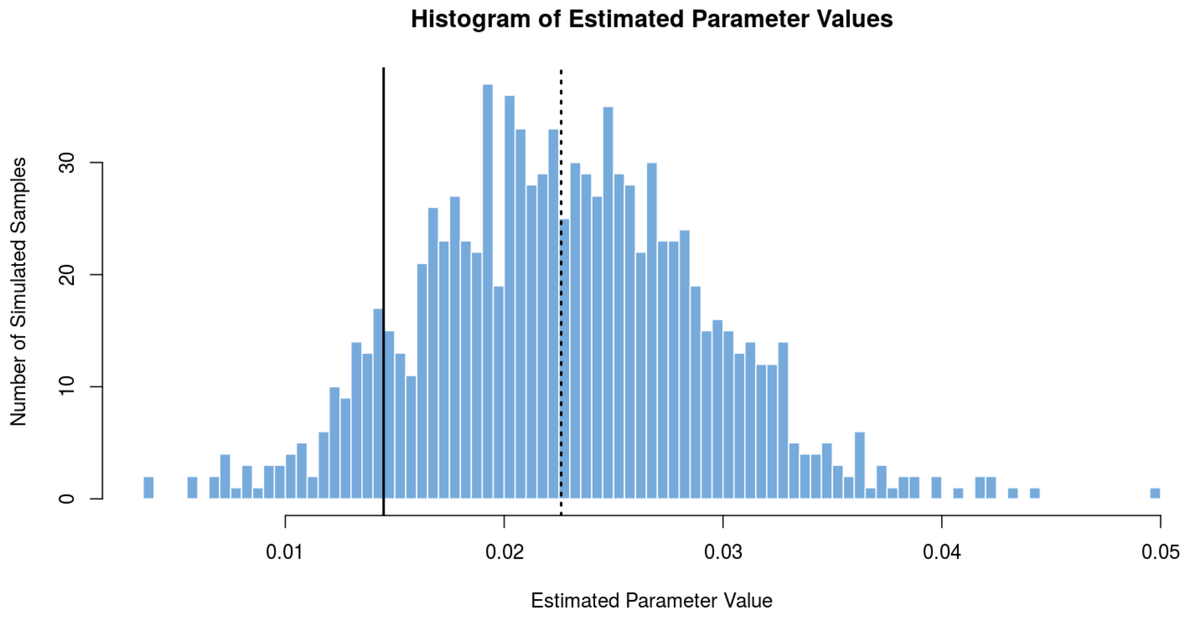

95% CI of estimates for pre-pregnancy weight (kg): 0.01, 0.04

Figure S19. Estimated parameter values for the coefficient of pre-pregnancy elevated blood pressure, structural model in the subsample in the MINA-Brazil Study.

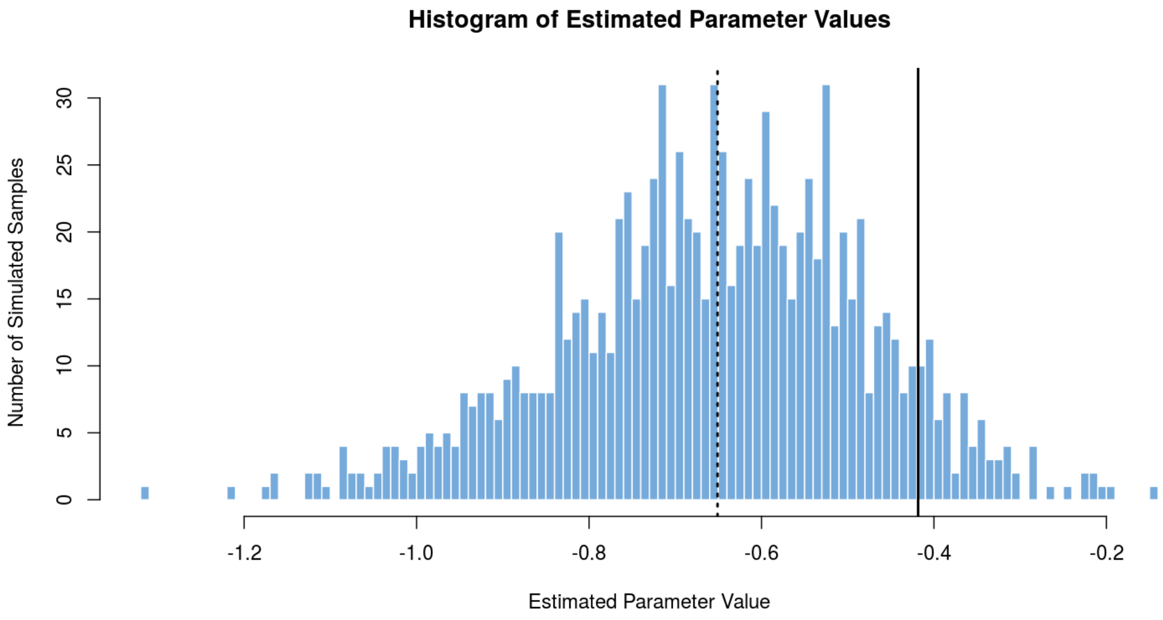

95% CI of estimates for pre-pregnancy elevated blood pressure: -1.03, -0.34

Figure S20. Estimated parameter values for the coefficient of primiparity, structural model in the subsample in the MINA-Brazil Study.

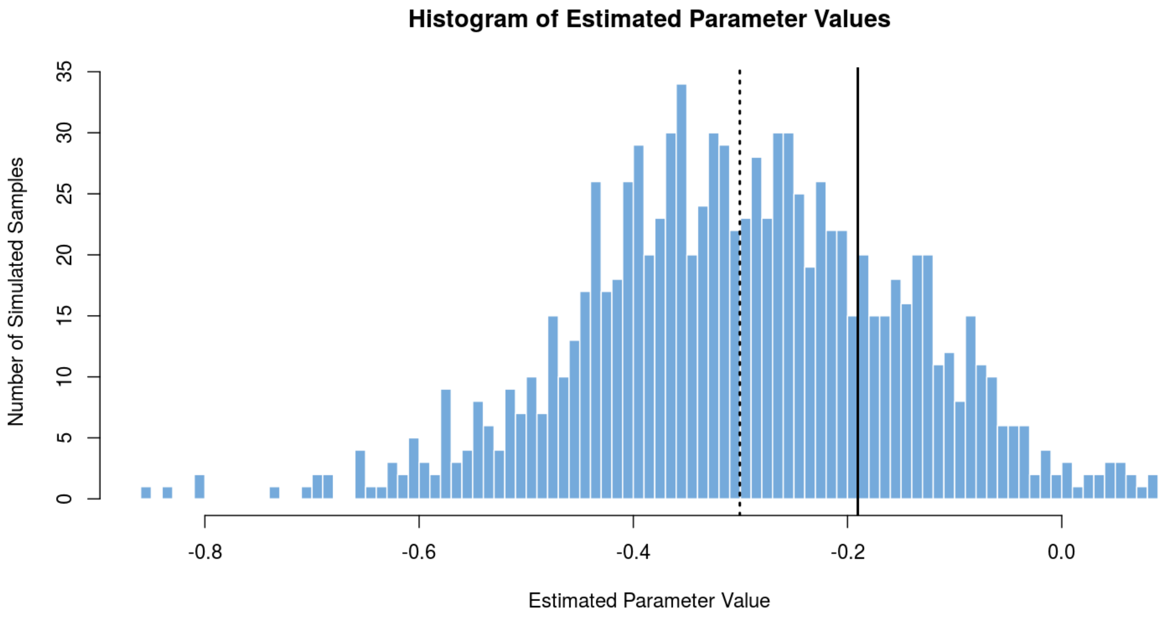

95% CI of estimates for primiparity: -0.60, -0.03

Figure S21. Estimated parameter values for the coefficient of smoking during pregnancy, structural model in the subsample in the MINA-Brazil Study.

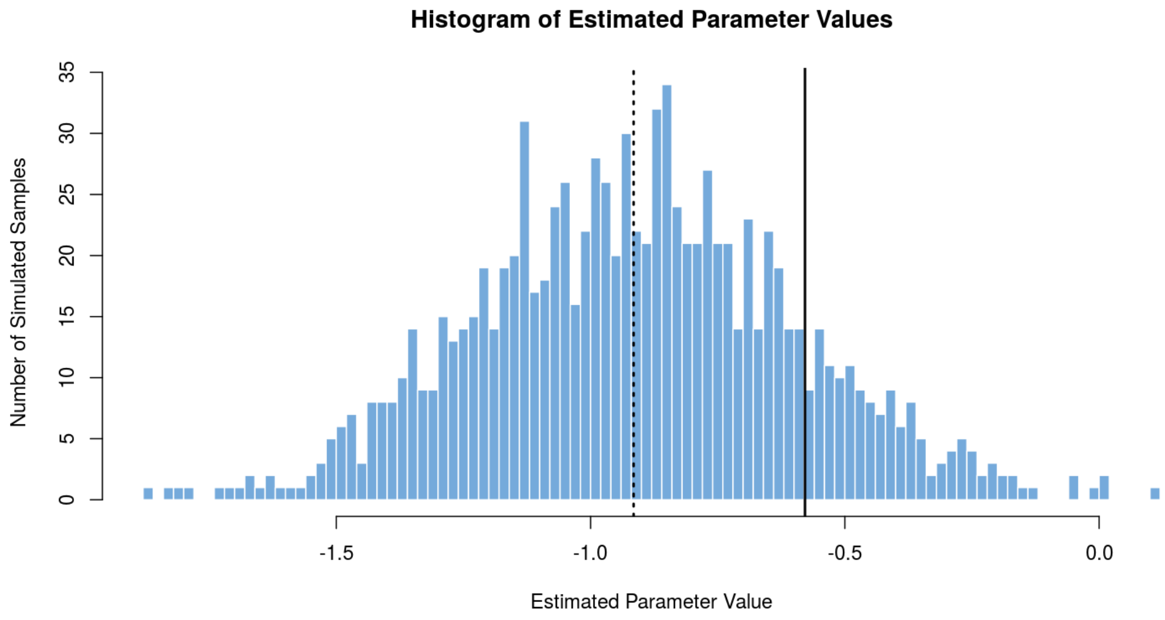

95% CI of estimates for smoking during pregnancy: -1.50, -0.28

Figure S22. Estimated parameter values for the coefficient of malaria during pregnancy, structural model in the subsample in the MINA-Brazil Study.

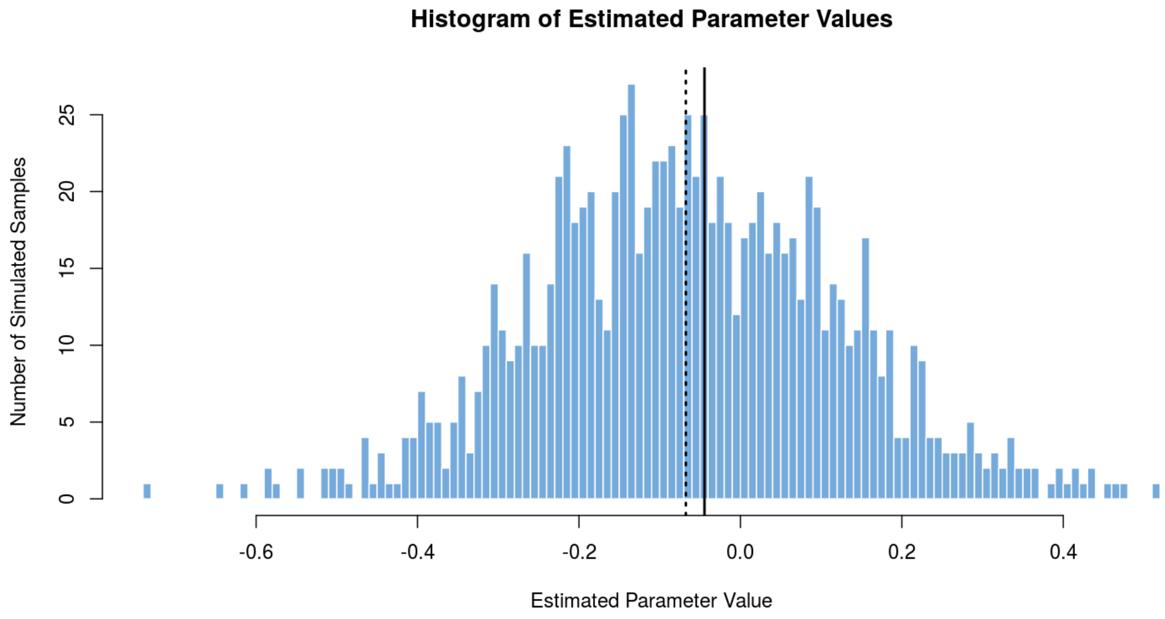

95% CI of estimates for malaria during pregnancy: -0.42, 0.32

Figure S23. Estimated parameter values for the coefficient of insufficient gestational weight gain, structural model in the subsample in the MINA-Brazil Study.

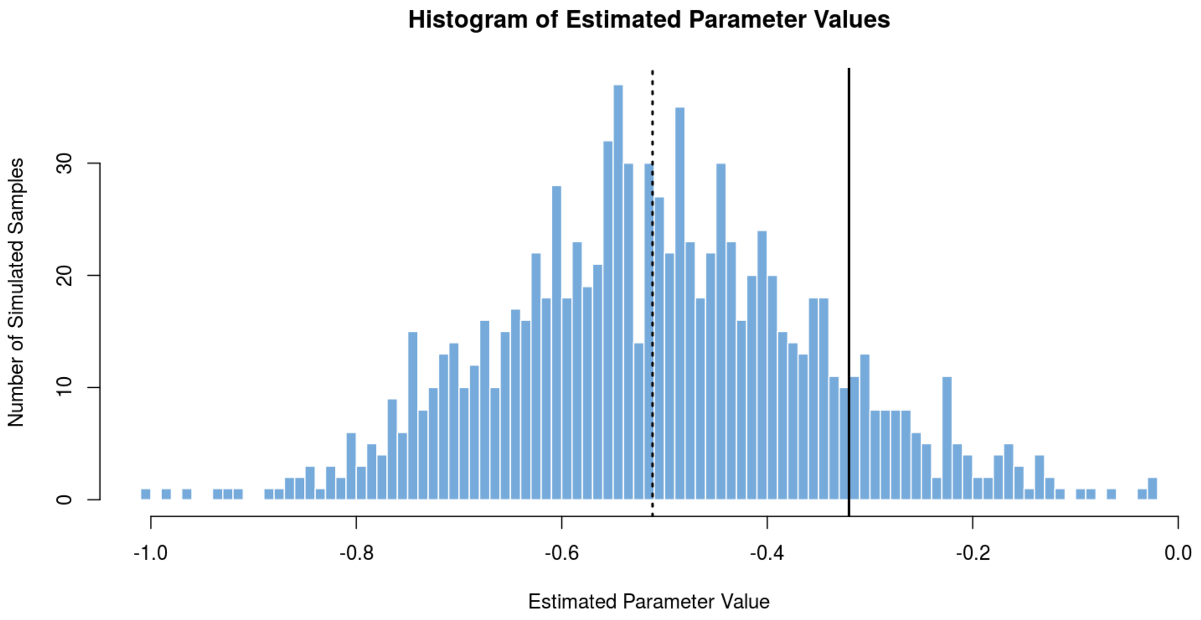

95% CI of estimates for insufficient gestational weight gain: -0.80, -0.18

Figure S24. Estimated parameter values for the coefficient of foetal head circumference (z-score), structural model in the subsample in the MINA-Brazil Study.

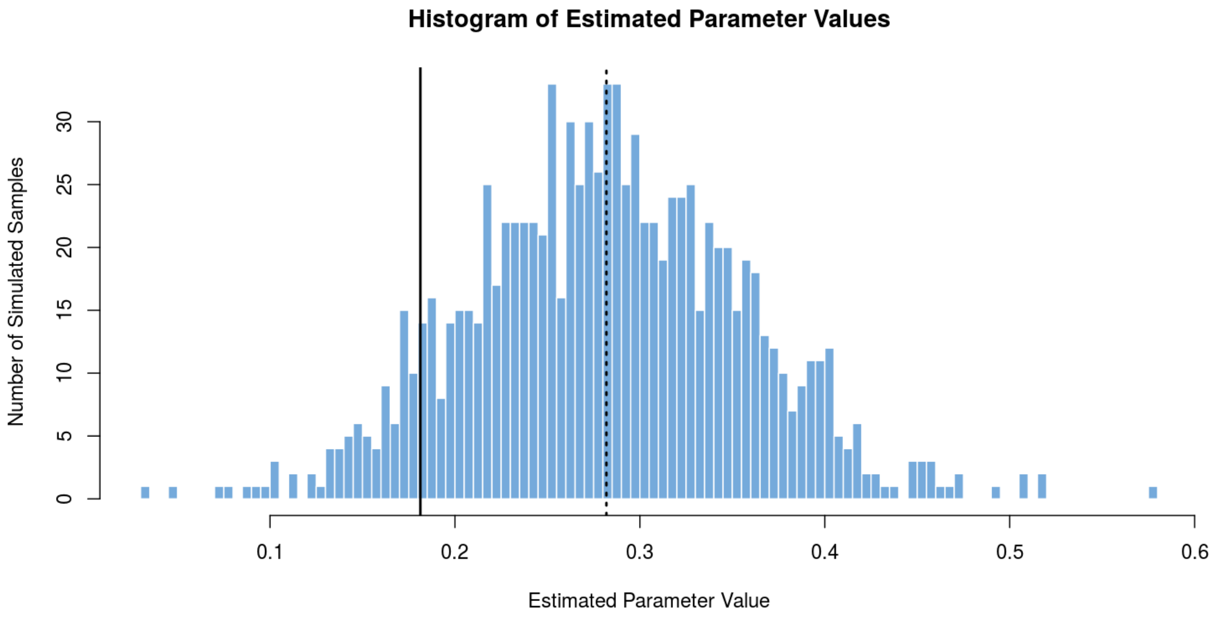

95% CI of estimates for foetal head circumference (z-score): 0.14, 0.42

Figure S25. Estimated parameter values for the coefficient of foetal abdominal circumference (z-score), structural model in the subsample in the MINA-Brazil Study.

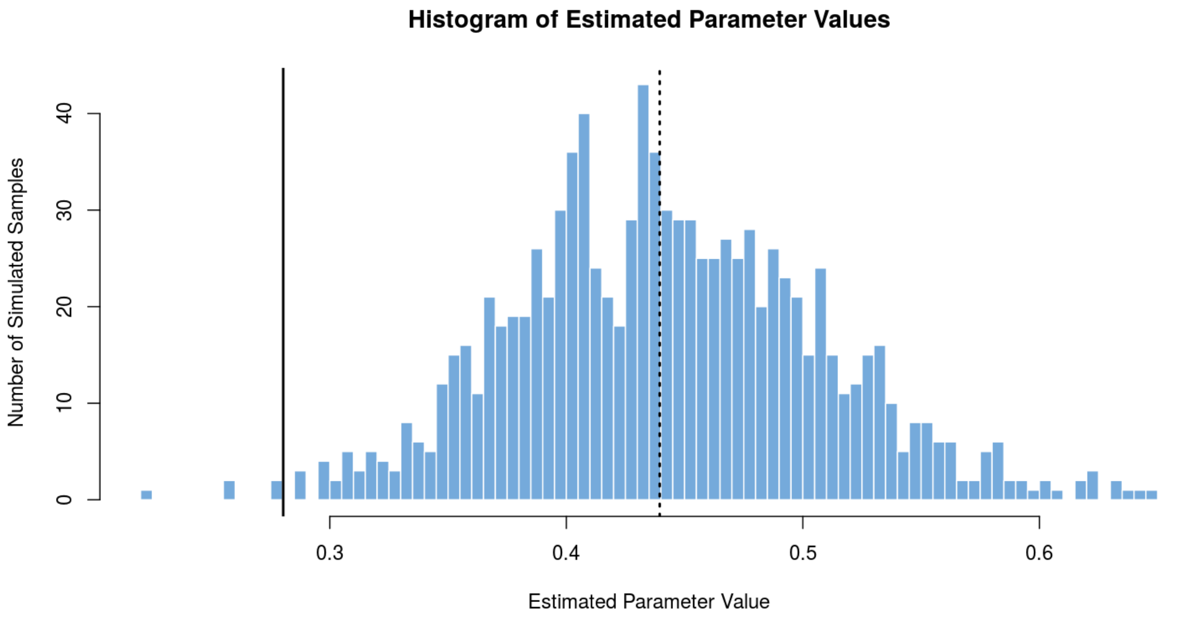

95% CI of estimates for foetal abdominal circumference (z-score): 0.32, 0.58

Figure S26. Estimated parameter values for the coefficient of foetal femur length (z-score), structural model in the subsample in the MINA-Brazil Study.

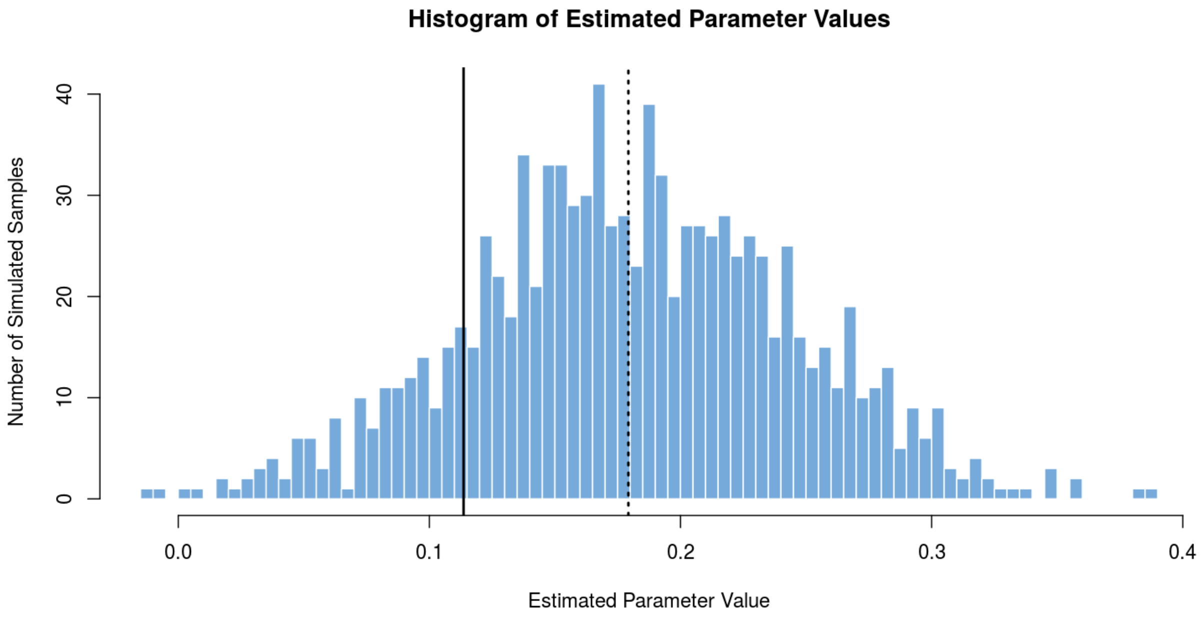

95% CI of estimates for foetal femur length (z-score): 0.05, 0.30
